# Synthesis and Characterization of β-Cyclodextrin-Essential Oil Inclusion Complexes for Tick Repellent Development

**DOI:** 10.3390/polym13111892

**Published:** 2021-06-07

**Authors:** Jennifer Hogenbom, Alysson Jones, Haozhe Vincent Wang, Laura Jane Pickett, Nicoletta Faraone

**Affiliations:** Department of Chemistry, Acadia University, Wolfville, NS B4P 2R6, Canada; 131226h@acadiau.ca (J.H.); alysson.jones@acadiau.ca (A.J.); 134932w@acadiau.ca (H.V.W.); 147241p@acadiau.ca (L.J.P.)

**Keywords:** terpenes, ticks, lemongrass, geranium Egyptian, lemon eucalyptus, cyclodextrin, repellent bioassay, SPME/GC-MS, encapsulation, inclusion complex

## Abstract

Essential oils (EOs) are used in several pest management applications. Due to their volatility, EOs may experience bioactivity reduction, thus requiring protection to extend their properties. In the present study, we investigated the inclusion complex formation (IC) of β-cyclodextrin (β-CD) with selected EOs with known tick repellent activity using two co-precipitation methods. ICs were characterized by evaluating EO mass concentration and inclusion efficiency (% IE) and other instrumental methods. Co-precipitation method 2 yielded the highest EO mass concentration (88 ± 6 μg/mg β-CD) for the 1:1 molar ratio geranium Egyptian EO IC. The EO volatile release over time from the ICs was investigated by headspace SPME/GC-MS analysis. ICs were also tested in tick repellency bioassays. ICs reported significant tick repellent activity, with lemongrass IC performing best overall. Method 1 showed the best combination of high mass concentration EO, controlled volatile release, and tick repellency with lemongrass EO. The results demonstrated that β-CD had selectively encapsulated different EOs. Moreover, the formation of ICs may improve EO tick repellent properties protecting the active ingredients and providing a better, long-lasting repellent action. These findings will allow the development of more effective naturally derived repellent products to protect individuals from tick bites and prevent tick-borne illnesses.

## 1. Introduction

Cyclodextrins (CDs) are cyclic, water-soluble oligosaccharides used in formulations since the 1970s to improve stability and delivery of active compounds in a wide range of applications, including food preservation and enhancement [1,2,3], drug delivery [4,5], and pest management and repellents [6,7]. CDs present 6–8 units of α-1,4-d-glucopyranose [5,8]. They have a conical shape with a hydrophilic outer surface and a hollow cavity slightly lipophilic in the middle. This shape allows the encapsulation of hydrophobic molecules providing protection from oxidation and UV degradation, as well as improving aqueous solubility [9,10]. In this regard, CDs have proven to be very useful in protecting essential oils (EOs): They form inclusion complexes (ICs) where hydrophobic EO components are encapsulated within the CDs hollow structure, protecting EO molecules while retaining their functional properties [11,12,13].

EOs have been targeted for use as natural product-based tick and insect repellents. Many plant-derived compounds found in EOs have shown repellency against ticks and other arthropods, providing suitable active ingredients [14,15,16] to be employed for the development of effective repellent products. The availability of natural product-based repellents provides important environmentally friendly alternatives to synthetic repellents. Concerns have been raised by many about the safety of synthetic repellents such as DEET for environmental and human health [17]. Repellents are a key method for preventing tick bites, which are responsible for the transmission of a variety of zoonotic diseases, including Lyme disease. Lyme disease presents a major public health concern, representing the most common vector-borne disease worldwide [18]. The main vector of the etiological agent of Lyme disease in North America is the blacklegged tick (*Ixodes scapularis*, Say 1821, Acari: Ixodidae), and rising average temperatures associated with climate change have been linked to the spread of *I. scapularis* ticks and the high incidence of Lyme disease cases [19,20,21].

EOs, such as lemongrass oil, and their main active molecules (such as citral and geraniol), have demonstrated significant tick repellent properties [14]. However, the repellency and stability of these compounds can be improved upon to achieve long-lasting effectiveness. An innovative approach is offered by exploiting the advantageous properties of CDs in encapsulating small, volatile, and unstable molecules. Though proven as effective repellents, EOs do have limitations due to their physical and chemical instability [22]. They are often highly volatile and prone to degradation in specific environmental conditions, requiring chemical manipulations (such as encapsulation) to improve their properties, duration of product, and efficacy.

The aim of this study was to synthesize β-CD ICs with selected EOs that have previously shown activity as tick repellents [14]. The ICs were synthesized according to two different methods and using three different active EOs as guest molecules, to provide information on the time effect on IC formation and chemical nature of guest molecules, and how these factors may impact chemical properties and the repellent activity of ICs. The ICs were assessed to determine (i) inclusion efficiency and quantity of the guest in β-CD, (ii) impact on volatile release rate, and (iii) repellency against ticks, specifically *I. scapularis* nymphs. 

## 2. Materials and Methods

### 2.1. Chemicals

Lemongrass (*Cymbopogon flexuosus*) and geranium Egyptian (*Pelargonium graveolens)* EOs were purchased from New Direction Aromatics (Mississauga, ON, Canada). Lemon eucalyptus (*Eucalyptus citriadora)* EO was purchased from Green Health (Lorton, VA, USA). Ethyl alcohol anhydrous (EtOH) was purchased from Commercial Alcohols (Greenfield Global, Brampton, ON, Canada). β-CD, hexane, tridecane, octanal, geranial, citronellal, and β-citronellol were purchased from Sigma-Aldrich (Oakville, ON, Canada).

### 2.2. Identification of Main Constituents of EOs

EO samples were analyzed via gas chromatography-mass spectrometry (GC-MS) to determine composition and main constituents. EOs were diluted in hexane (CHROMASOLV, >98.5% purity, Sigma-Aldrich, St. Louis, MO, USA) at the concentration of 50 ng/μL. Samples were analyzed using a Scion 456 Gas Chromatograph-Single Quad (GC-MS; SCION Instruments UK Ltd., Livingston, UK). A non-polar capillary column Rxi®-5silms (30 m × 0.25 mm, film thickness 0.25 mm; Restek Corporation, State College, PA, USA) linked to a Bruker mass spectrometer (Bruker Daltonics Ltd., Coventry, UK) was used for analysis. Samples underwent splitless injection and the oven temperature was programmed for 5 min at 50 °C then increased to 186 °C at 8 °C/min, and then increased to 280 °C at 30 °C/min held for 5 min. Both the injector and transfer line were maintained at 250 °C. Helium was used as a carrier gas. Compounds were identified using analytical standards and NIST Mass Spectral Search Program for the NIST/EPA/NIH Mass Spectral Library version 2.0 g 2011 (Scion Instruments UK Ltd., Livingston, West Lothian, UK).

### 2.3. Preparation of Essential Oil ICs

ICs of three EOs in β-CD were prepared by two co-precipitation methods [1,6]. Encapsulated products were prepared based on a 1:1 molar ratio of β-CD to the main component present in the EO composition. Ethanol was used as a co-solvent. A summary of the prepared ICs is reported in Table 1.

#### 2.3.1. Method 1

Initially, 0.0088 mol of β-CD was added to 100 mL of 1:2 ethanol:water and heated at 50–60 °C while stirring until fully dissolved. The β-CD solution was then cooled down to 30 °C. Then, 0.0088 mol of EO as determined using the main constituents was dissolved in EtOH at 10% *v*/*v*. The EO solution was added dropwise to the β-CD solution and stirred for 30 min. The precipitate formed was collected by suction filtration and left under vacuum for 1.5 h to dry. The product was then further dried in an oven at 50 °C for 1.5 h before being transferred to a glass vial and placed in a desiccator for storage until further use [6].

#### 2.3.2. Method 2

Initially, 0.0088 mol of β-CD was mixed in 100 mL of 1:2 ethanol:water and heated at 50–60 °C while stirring until fully dissolved. An equivalent molar amount (0.0088 mol) of EO determined from the main constituents was dissolved in EtOH at 10% (*w*/*v*). While maintaining stirring and heating of the β-CD solution, the EO solution was added dropwise. The mixture was left to stir for an additional 4 h, covered without heating. The mixture was stored overnight at 4 °C before collecting the precipitate by vacuum filtration and drying in an oven at 50 °C for 24 h before being transferred to a glass vial and placed in a desiccator for storage until further use [1].

#### 2.3.3. Physical Mixture

A physical mixture of EO and β-CD was prepared in water for UV analysis. The amount of both were determined based on the required amount of EO required to obtain an absorbance of around 1 in the EO alone. The desired amount of EO and β-CD were each weighed into a 25 mL volumetric flask and diluted in water.

### 2.4. GC-FID Quantificaiton of EO in ICs

The mass concentration and inclusion efficiency (*IE*) of the EO in the ICs was quantified by GC-FID analysis [11]. The % IE of the EO in the prepared ICs represents the percent of EO recovered into the final product and is calculated as follows:

$$\left(\%\;\mathrm{IE}\right)=\frac{\;\mathrm{Mass}\;\mathrm{EO}\;\mathrm{in}\;\mathrm{IC}}{\mathrm{Initial}\;\mathrm{mass}\;\mathrm{EO}\;\mathrm{to}\;\mathrm{be}\;\mathrm{encapsulated}}$$

The EO was extracted from the ICs by hexanes extraction prior to GC quantification.

#### 2.4.1. Extraction of EO

Extractions were done in triplicate. Ten (10) mg of IC was dissolved in 1 mL of deionized H_2_O. Then, 1 mL of 1:1 hexane/ethanol was added, and the solution was shaken manually. Finally, 1 mL of hexane was added, and the solutions were sonicated for 30 min at room temperature and 40 kHz in an ultrasonic bath. The organic layer was collected, and the aqueous layer was extracted twice more with 1 mL of hexane. Organic layers were combined (final volume was 3.5 mL), dried with Na_2_SO_4_, and filtered using cotton wool.

#### 2.4.2. GC-FID Analysis of EO Extracts

Extracts were diluted by half with 80 ng/μL internal standard solution (final internal standard concentration 40 ng/μL) and analyzed by GC-FID. The internal standard used for LE and LG complexes was tridecane, while octanol was used as an internal standard for GE complexes. Each extract was analyzed on a gas chromatograph (Scion 450-GC; SCION Instruments UK Ltd., Livingston, UK) equipped with a flame ionization detector (FID). The GC-FID was equipped with a Rxi®-5silms capillary column (30 m × 0.25 mm, film thickness 0.25 mm; Restek Corporation, State College, PA, US). The oven was programmed to start at 50 °C for 5 min, followed by a heating ramp of 8 °C/min to 186 °C, followed by 30 °C/min until 280 °C, where it was held for 5 min. The carrier gas was Helium at a flow rate of 1.20 mL/min. The total analysis time was 30.13 min. The detector was set at 320 °C. The injector temperature was 250 °C, 1 μL of the sample was injected manually with a split ratio of 1:20. Quantification of the EO content was done using a 400–10 ng/μL standard curve containing 40 ng/μL internal standard in hexanes. The peak from the main constituents of the EO was normalized using the internal standard peak for quantification. The mass concentration of the EO in the IC was calculated along with the % IE.

### 2.5. SPME/GC-MS Quantification of EO Volatiles Over Time

The release of EO volatiles from ICs were quantified using GC-MS by solid phase micro extraction (SPME) analysis [23,24]. The volatiles released were measured after 0, 3, 6, 9, 18, and 24 h of exposure at room temperature and 10–20% relative humidity. This was done by placing ICs on a watch glass stored in a desiccator for the allotted time. Calcium carbonate was used as a desiccant. After the allotted time had passed, 10 mg of sample was weighed into a 20 mL headspace vial (Gerstel, Linthicum, MD, US), sealed with PTFE thread tape, and capped. Five (5) mg were used for quantification of LE-1 and LE-2 due to their high amount of volatile release. Released EO volatiles were quantified using liquid standards. The SPME analysis was performed using a Scion SQ GC-MS equipped with a Gerstel multipurpose autosampler (MPS) (Gerstel, Mülheim an der Ruhr, Deutschland). The MPS allowed for automated SPME using a PDMS SPME fiber (100 μm). The SPME fiber was conditioned at 250 °C for 5 min before exposure to the sample. The samples in headspace vials were incubated for 10 min at 35 °C in the MPS agitator set at 250 rpm with an interval of 5 s on and 2 s off. The SPME fiber was exposed to the sample for 5 min with the same heating and stirring program. The SPME fiber was then desorbed in the GC inlet for 180 s before being reconditioned at 250 °C for 30 min. The same GC program described previously was used with a carrier gas flow rate of 1.00 mL/min and split delay of 1 min. LE samples were analyzed with higher initial split ratio of 1:50 to account for high volatile release. The liquid standard curve was quantified using the same GC-MS method in triplicate with 1 μL liquid injections using Gerstel MPS.

### 2.6. FT-IR Analysis of ICs

Fourier-transform infrared spectroscopy (FT-IR) was used to characterize the ICs. The spectra of the EO and β-CD alone were also obtained for comparison. The IC and β-CD spectra were obtained using KBr pressed pellets, while the spectra of the EOs were obtained using KBr plates. The KBr pellets were prepared by mixing the ICs with KBr powder in a ratio of ~1:10 and grounding in a mortar and pestle. The homogenized powder was then pressed into a disk with a hand press for ~2 min. The EO spectra were obtained by spreading a thin layer of the oil between polished KBr plates. The FT-IR spectra were obtained in the range of 400–4000 cm^−1^ with a resolution of 3.857 cm^−1^ from an average of 32 scans on a FT-IR spectrometer (Nicolet Avatar 360, Nicolet Instrument Corporation, Danbury, CT, USA).

### 2.7. Nuclear Magnetic Resonance (NMR) Spectroscopy Analysis

The ICs and β-CD were analyzed by ^1^H-NMR spectroscopy on a Bruker Avance Neo 400 MHz spectrometer (Billerica, MA, USA) in D_2_O to determine if the desired EO:β-CD IC was formed. Chemical shifts are relative to internal TMS. NMR data processing was done using TopSpin 4.0.8 (Bruker BioSpin, 2019).

### 2.8. UV-Vis Spectroscopy

The UV-Vis absorbance spectra of the ICs were measured in DI H_2_O from 400 to 190 nm. The spectra of EO alone, β-CD alone, and a physical mixture (PM) of β-CD and EO were also measured. The concentration of EO solution was adjusted to obtain initial maximum absorbance around 1. All subsequent solutions were prepared with this EO concentration. To obtain the desired concentration of EO in the IC solutions the mass concentration of EO, as determined by GC-FID analysis, was used. The concentration of β-CD in the PM and β-CD alone was approximately the same as that in the IC solution. The absorbance spectra of each solution were measured 24 h after preparation in quartz cuvettes with a path length of 1 cm on a Cary 100 Bio spectrophotometer (Varian, Palo Alto, CA, USA).

### 2.9. Scanning Electron Microscopy (SEM)

The ICs were examined by scanning electron microscopy (SEM). Prior to analysis, the samples were mounted on SEM cylindrical specimen mounts using adhesive tabs. These mounted samples were then coated with gold and palladium using a Polaron SC7640 Sputter Coater (Quorum Technologies, Lewes, UK) under automatic coating methods (Quorum Technologies, 2008). The prepared samples were observed and photographed using a JEOL LV-5900 scanning electron microscope (JEOL USA, Peabody, MA, USA) located at the Acadia Centre for Microstructural Analysis (ACMA) at Acadia University.

### 2.10. Tick Repellency Bioassays

#### 2.10.1. Vertical Bioassay

**Figure 1:**
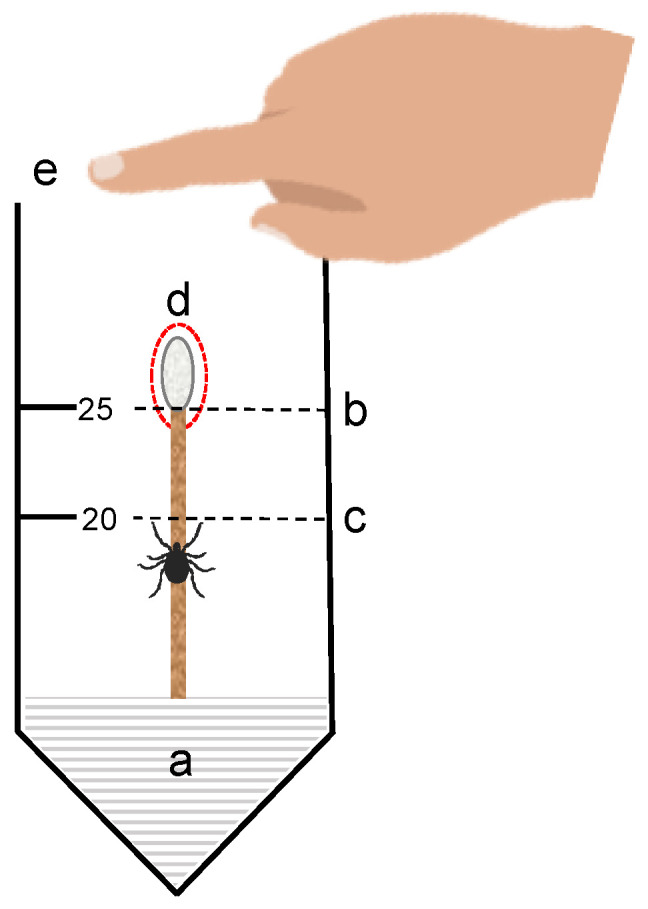
Vertical bioassay setup: A cotton swap is inserted in a Falcon tube and secured at the base with a polystyrene platform (**a**). The base of the cotton swap tip is aligned with the 25 mL line (**b**); the tick is released at the bottom of the cotton tip just below the 20 mL line. The timer starts when the tick crosses the 20 mL line (**c**); the treatment is applied on top of the swab (**d**); the observer exposes a finger on the open top of the Falcon tube to stimulate the tick to climb the slender surface (**e**).

Vertical bioassays (Figure 1) were prepared with modifications to a previously described vertical bioassay method [25]. Naïve, unfed, host-seeking (actively questing), *I. scapularis* ticks were used in all behavioural assays. Uninfected ticks were purchased from the Tick Rearing Facility Laboratory at Oklahoma State University (Stillwater, OK, USA). Ticks were stored on site in plastic containers lined with moistened Kimwipe® at 4 °C in dark conditions.

Nymphs were removed from the refrigerator and kept at room temperature for approximately 20 min prior to starting the bioassays. The bottom of 50 mL Falcon tubes (Fisher Scientific, Walthman, MA, USA) were filled with polystyrene foam. Cotton swabs (SolonCare^®^, Amd-Ritmed Inc., Montréal, QC, Canada) were measured and cut at 7 cm so that the edge cotton tip was at the 25 mL marker on the falcon tube when fully inserted. In addition, 100 mg/mL solutions of ICs were prepared in 1:1 DI H_2_O:EtOH for use in the assay. Fifty (50) µL of the solution was pipetted to the tip of the cotton swab and left to dry for approximately for 5 min. Active ticks were added with a paintbrush to the wooden stem of the cotton swab just below the 20 mL marker on the tube and positioned so that the tick faced upward. Time was started when the tick crossed the 20 mL marker. Time was stopped when the tick either reached the tip and remained for 10–15 s, reached the tip, and dropped or reached the tip and climbed off the tip. The observer placed their hand on the opening of the tube for the length of the experiment and occasionally removed their hand to breath over the top of the tube.

#### 2.10.2. Statistical Analysis

Statistical analyses were performed using RStudio version 1.1.453 (RStudio Team 2018). Results from the mass of EO extracted from IC and EO volatile emission were analyzed with a linear mixed-effect model (lmer). The significance of the model was assessed by running the anova function. We performed a post-hoc pairwise comparison of interaction using the emmeans function (emmeans package) on the model to determine the differences between means produced by different EOs. Results from different methods and time points were organized in subsets and independently analyzed. Repellency data with not normal distribution were subjected to non-parametric tests (i.e., Kruskal-Wallis), followed by a post-hoc test (i.e., Dunn test) to compare the different treatments. Differences were considered significant at *p* ≤ 0.05.

## 3. Results

### 3.1. Main Constituents of Essential Oil

**Figure 2:**
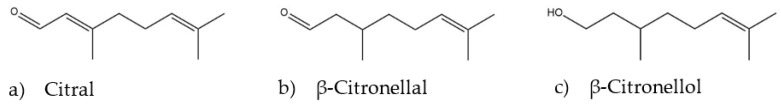
Structure of main components found in selected essential oils. (**a**) Citral, present in lemongrass oil; (**b**) *β*-citronellal, present in lemon eucalyptus oil; and (**c**) *β*-citronellol, present in geranium Egyptian oil.

The main constituents of EOs were determined by GC-MS analysis. Citral, *β*-citronellal, and *β*-citronellol were identified as the major components of LG, LE, and GE EOs, respectively (Figure 2). The response from these compounds was used in the various GC quantification methods for the respective ICs. 

### 3.2. GC-FID Quantificaiton of EO in ICs

The mass concentration and % IE of essential oil in the products was determined from the GC-FID analysis. The results of this analysis are included in Table 2. The mass concentration and the % IE of LG in the inclusion complex were comparable for the two methods, 65 ± 2 μg/mg and 65 ± 2% for method 1, and 63 ± 3 μg/mg and 69 ± 4% for method 2. On the other hand, the amount of GE oil, extracted from the complex prepared according to method 2, was significantly higher (88 ± 6 μg/mg) compared to method 1 (73 ± 5 μg/mg), while % IE was similar for both methods. Overall, LE was the guest that showed the least retained within β-CD, providing a low mass concentration (30 ± 1 μg/mg for method 1, 24 ± 1 μg/mg for method 2) and a low % IE (25 ± 1% for method 1, 20 ± 1% for method 2) compared to the other guest EOs.

### 3.3. Volatile Release Over Time

**Figure 3:**
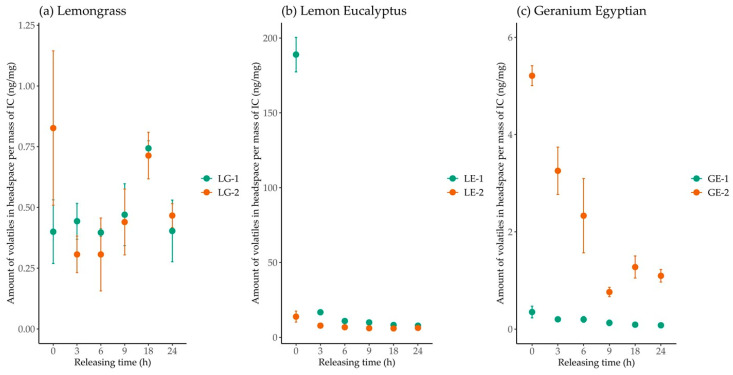
Essential oil (EO) volatiles from (**a**) lemongrass:β-cyclodextrin, (**b**) lemon eucalyptus:β-cyclodextrin, and (**c**) geranium Egyptian:β-cyclodextrin inclusion complexes released over time. Volatiles measured by SPME/GC-MS (N = 3). Quantification was done using liquid standard curves of corresponding EO in hexanes. The results compare the two inclusion complex preparation methods for each guest EO.

The release of EO volatiles from the ICs was quantified at 0, 3, 6, 9, 18, and 24 h by SPME/GC-MS (Table 3). LG:β-CD complexes prepared with the two methods presented a similar release pattern (Figure 3a). LG-1 maintained a constant release of volatiles (0.40 ± 0.10 ng/mg), aside from an increase at 18 h. Overall, the volatile release from LE and GE ICs were not consistent overtime, with a significant decrease in volatiles released after 0 h. The LE:β-CD complex prepared with method 1 was found to have the greatest amount of volatiles released (189 ± 9 ng/mg) at 0 h. 

### 3.4. FT-IR

**Figure 4:**
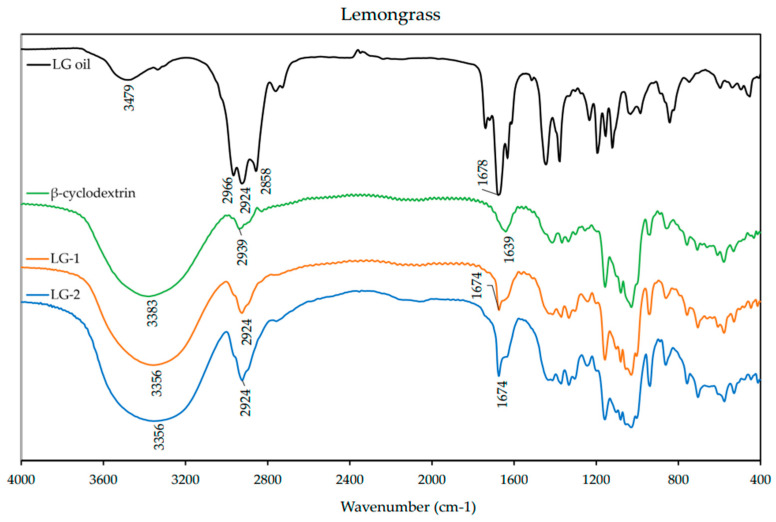
FT-IR spectra of lemongrass (LG) essential oil, β-cyclodextrin, and inclusion complexes LG-1 and LG-2. Spectra were obtained from 400–4000 cm^−1^ with a resolution of 3.857 cm^−1^ as an average of 32 scans on a FT-IR spectrophotometer.

**Figure 5:**
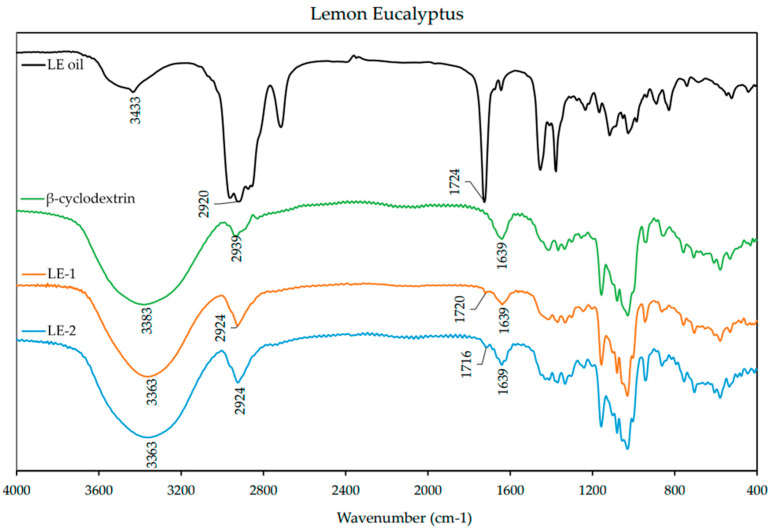
FT-IR spectra of lemon eucalyptus (LE) essential oil, β-cyclodextrin, and inclusion complexes LE-1 and LE-2. Spectra were obtained from 400–4000 cm^−1^ with a resolution of 3.857 cm^−1^ as an average of 32 scans on FT-IR spectrophotometer.

**Figure 6:**
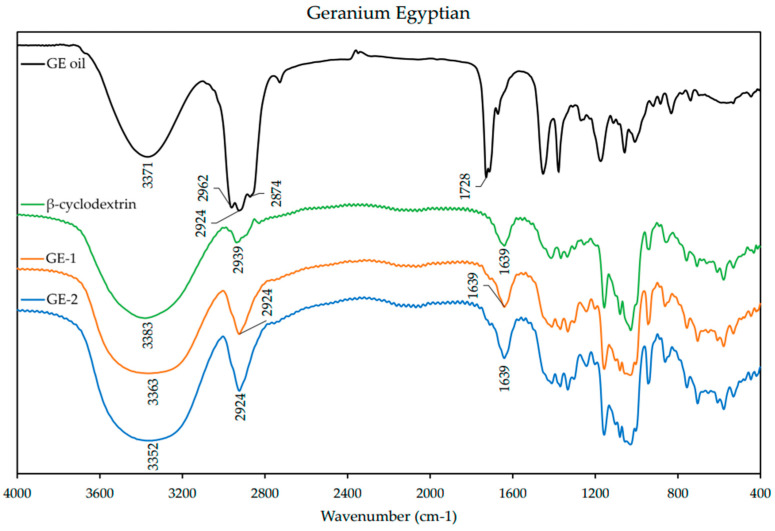
FT-IR spectra of geranium Egyptian (GE) essential oil, β-cyclodextrin, and inclusion complexes GE-1 and GE-2. Spectra were obtained from 400–4000 cm^−1^ with a resolution of 3.857 cm^−1^ as an average of 32 scans on an FT-IR spectrophotometer.

FT-IR spectra were obtained from individual EO, β-CD, and ICs. The spectra are presented below for each EO and its respective ICs in Figure 4, Figure 5 and Figure 6. The spectrum of β-CD is included in each figure for comparison. A summary of the FT-IR data is included in the Appendix A (Table A2).

The spectrum of LG (Figure 4) presented prominent absorption bands of stretching vibration (3479 cm^−1^) and scissoring vibration (1446 cm^−1^) of =CH, asymmetrical and symmetrical stretching vibrations (2966–1858 cm^−1^) of –CH_2_ and stretching vibration of C=O (1678 cm^−1^). The spectrum of β-CD displayed prominent absorption bands at 3383 cm^−1^ for O–H stretching vibration, 2939 cm^−1^ for –CH_2_ asymmetrical stretching vibration, 1639 cm^−1^ for H–O–H bending vibration, and 1157 cm^−1^ for C–O–C asymmetrical stretching vibration. The spectra of the ICs presented the characteristic absorption band of aldehyde C=O at 1674 cm^−1^ exhibiting a weaker intensity and lower frequency than the LG alone. However, there was no other feature similar to LG EO alone when examining the spectra of ICs.

The spectrum of LE (Figure 5) presented many similarities with the one of LG EO. A prominent absorption band of stretching vibration (3443 cm^−1^) and scissoring vibration (1450 cm^−1^) of =CH, asymmetrical and symmetrical stretching vibrations (2920 cm^−1^) of –CH_2_, and stretching vibration of C=O (1724 cm^−1^) associated with the aldehyde group in the main component (citronellal). The spectra of the ICs exhibited changes in features compared to the free EOs, stretching vibration of C=O was present at lower frequencies, and intensity and asymmetrical and symmetrical stretching vibrations (2920 cm^−1^) of –CH_2_ was present at a much lower intensity and slightly different frequency. 

The GEs spectrum (Figure 6) presented the typical prominent absorption bands at 3371 cm^−1^ for O–H stretching vibration (from geraniol main component), scissoring vibration (1450 cm^−1^) of =CH, asymmetrical and symmetrical stretching vibrations (2962–2874 cm^−1^) of –CH_2_, and stretching vibration of C=O (1728 cm^−1^). Similarly, the spectra of both ICs presented different features than the free EOs. Stretching vibration of C=O at 1728 cm^−1^ was indistinguishable and asymmetrical and symmetrical stretching vibrations (2962–2874 cm^−1^) of –CH_2_ are present at a much lower intensity and slightly different frequency.

### 3.5. NMR

**Figure 7:**
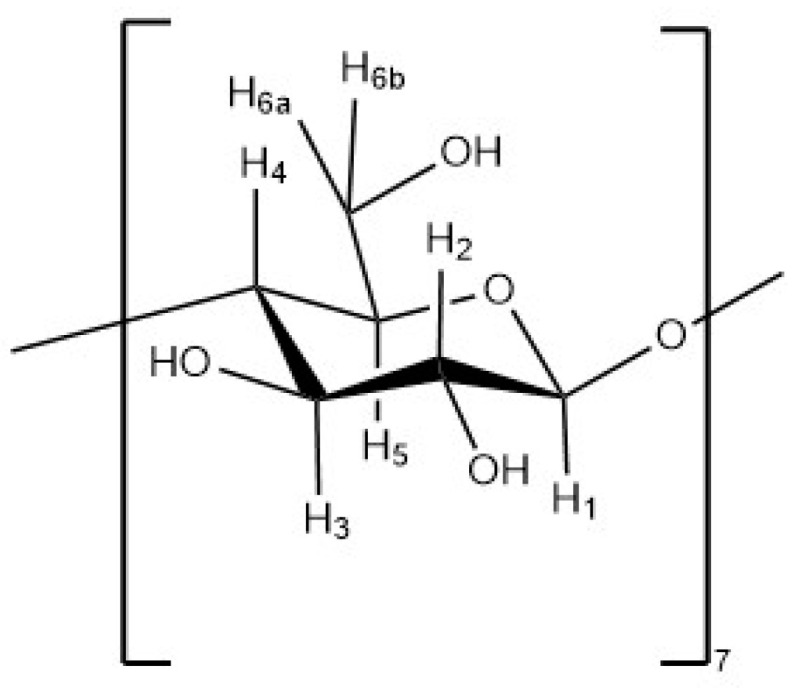
Structure of the repeating unit of β-CD. Protons are labeled according to their assignment for the NMR analysis.

The spectrum of β-CD was compared to the spectrum of the ICs. The H-3 and H-5 protons of β-CD are located on the interior of the inclusion complex of essential oil and β-CD. It has been reported that the containment of a guest in the β-CD will affect the chemical shifts of these protons [11,26]. The structure of the repeating unit of β-CD is depicted in Figure 7. 

From the NMR analysis, a significant difference in chemical shifts between the ^1^H-NMR spectra of β-CD and ICs was found with respect to proton 3 and 5. A summary of the NMR data is presented in Table 4 and Table A1.

### 3.6. UV-Vis Spectroscopy

**Figure 8:**
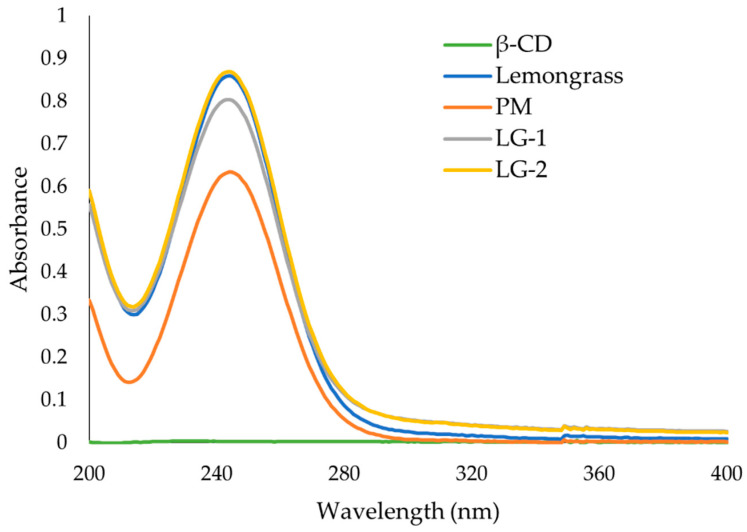
UV-Vis spectra of β-cyclodextrin (β-CD), lemongrass (LG) essential oil, physical mixture of both (PM) and the inclusion complexes LG-1 and LG-2. Solutions were prepared in water and have the same β-CD or essential oil concentration where applicable.

**Figure 9:**
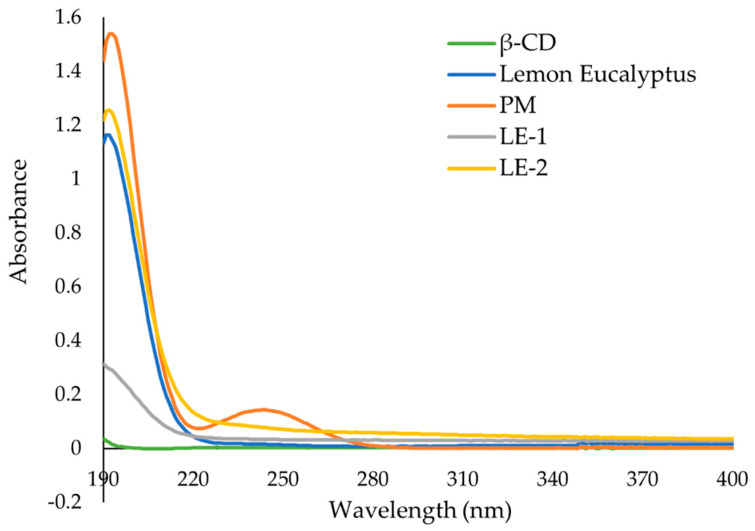
UV-Vis spectra of β-cyclodextrin (β-CD), lemon eucalyptus (LE) essential oil, physical mixture of both (PM) and the inclusion complexes LE-1 and LE-2. Solutions were prepared in water and have the same β-CD or essential oil concentration where applicable.

**Figure 10:**
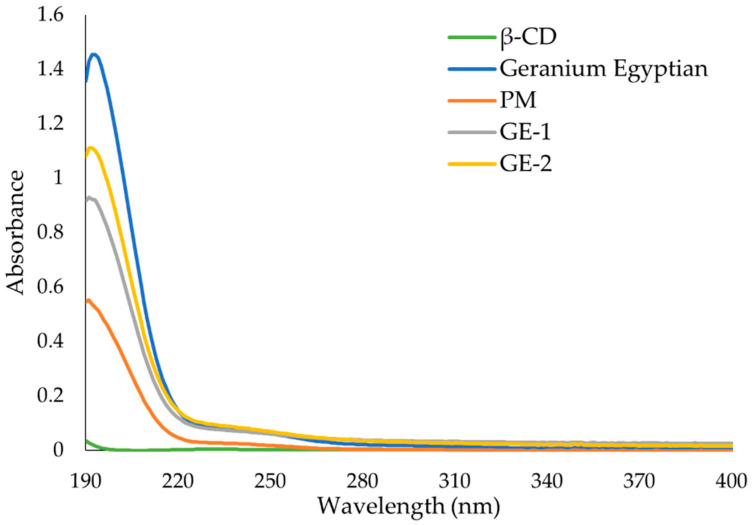
UV-Vis spectra of β-cyclodextrin (β-CD), geranium Egyptian (GE) essential oil, physical mixture of both (PM) and inclusion complexes GE-1 and GE-2. Solutions were prepared in water and have the same β-CD or essential oil concentration where applicable.

The UV-Vis absorbance spectra of each essential oil, β-CD, and the ICs are presented in Figure 8, Figure 9 and Figure 10.

LG EO has a maximum absorbance at 244 nm (Figure 8). This peak at 244 nm is also found in LG EO and β-CD physical mixture (PM) at a reduced absorbance. The spectra of LG-1 and LG-2 both have the same peak at 244 nm which exhibit higher absorbance than in the PM at the same concentration. LG-2 had comparable maximum absorbance to the LG EO alone.

As seen in Figure 9, LE EO has maximum absorbance at 191 nm. The same peak is found in the PM at higher absorbance. The high absorbance recorded for the physical mixture was not observed in the other EOs, and this may be related to the spontaneous formation of IC in the solution when the UV spectra were acquired [11,12]. The spectra of LE-2 had the same peak at 191 nm which exhibits higher absorbance than EO alone. LE-1 has lower absorbance than both PM and LE-2. This phenomenon was not observed for the other ICs and may be due to poor IC formation. 

As seen in Figure 10, GE has maximum absorbance at 191 nm. This peak is also found in the physical mixture (PM) of essential oil and β-CD at reduced absorbance. The spectra of GE-1 and GE-2 had the same peak at 191 nm which exhibits lower absorbance than EO alone at the same concentration. GE-1 and GE-2 do however have higher absorbance than the PM of EO and β-CD.

### 3.7. SEM

**Figure 11:**
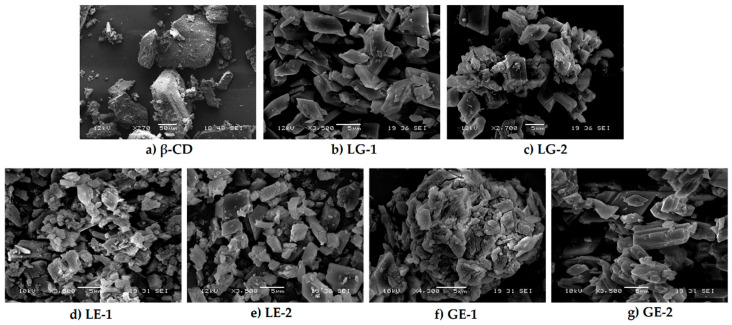
SEM images of (**a**) β-cyclodextrin (β-CD); β-CD inclusion complexes with lemongrass oil (**b**) LG-1 and (**c**) LG-2; inclusion complexes with lemon eucalyptus oil (**d**) LE-1 and (**e**) LE-2; inclusion complexes with geranium Egyptian oil (**f**) GE-1 and (**g**) GE-2.

Photographs of β-CD and all prepared ICs were taken under a scanning electron microscope to examine particle morphology (Figure 11). The sample of β-CD exists in an amorphous, irregular crystal [27]. However, the ICs appear in lamellate morphology. The comparison of the images reveals that the ICs are structurally distinct from β-CD, also in terms of size: β-CD particles range up to 50 μm, while IC particles are around 5 μm.

### 3.8. Tick Repellency Bioassay

Results of the repellency bioassay are summarized in Table 5. Ticks were significantly repelled by the encapsulated oils prepared by both methods (method 1, χ^2^ = 14.82, df = 3, *p* = 0.002; method 2, χ^2^ = 20.31, df = 3, *p* = 0.0001). LG ICs prepared with both methods exerted up to 80% (Z = −3.53, *p* = 0.002, method 1; Z = −3.53, *p* = 0.001, method 2) of tick repellency, while GE IC prepared according to method 2 was overall the most effective in repelling ticks up to 90% (Z = −3.61, *p* < 0.001). Among the ICs, LE IC prepared according to method 1 was the least effective, repelling only 50% (Z = −2.21; *p* = 0.05) of tested ticks.

## 4. Discussion

Beta-cyclodextrin resulted as an effective host in encapsulating selected EOs with known repellent activity. The tested oils showed varying levels of success in forming a stable IC [13]. The two investigated co-precipitation methods influenced the inclusion efficiency, amount of volatiles released, and the complexes ability to repel ticks [6]. In addition to the method variation, a different affinity with the studied EOs in forming the complex is reported. Lemongrass essential oil resulted in being the most compatible at forming IC with both methods. The essential oil used in this study has citral as a primary constituent [28], which may account for about 75% of the overall composition, providing a more homogeneous guest material. LG-1 and LG-2 were found to contain an equivalent mass concentration of EO and % IE. The LE ICs retained the least EO of the three tested inclusion complexes, with 25 and 20% IE for methods 1 and 2, respectively. GE EO was encapsulated the most successfully using method 2, obtaining the highest mass concentration of EO overall. The differences in the prepared ICs between the method and EO support other findings [29,30] that size and chemical properties of guest molecules significantly impact inclusion efficiency and ability of CDs to retain the compound(s) in the host cavity. The shorter method (method 1) may be more suited for commercial production. On the other hand, although the longer method (2) requires more time for encapsulation to occur, it may improve inclusion efficiency, and the longer drying time may reduce the amount of non-encapsulated EO on the IC surface, creating a product which has a more consistent essential oil release.

The release of EO volatiles over time from the ICs was characterized by SPME/GC-MS analysis. In previous studies, the controlled release from CD ICs has often been measured by static or multiple headspace extraction [9,31]. Our approach of using SPME/GC-MS allows small concentrations of volatile molecules present in the headspace to be detected and quantified. The different preparation methods influenced the release of EO volatiles over time from the ICs, revealing a variation between the different EOs encapsulated [6,32,33]. Volatiles released from LG-1 were not significantly different over the 24 h, with an exception at 18 h where the release doubled. The mostly consistent release across 24 h indicates that method 1 was most successful at forming a stable inclusion complex of LG and β-CD to control the EO volatile release over time. Method 1 yielded the greatest initial volatile release from the LE IC, which decreased drastically over 24 h. Method 2 exhibited a less drastic change of volatile release for LE, proving more successful at retaining the LE EO in the cyclodextrin cavity. ICs containing GE also showed decreases in volatile release over time. The amount of initial GE volatiles released from the two treatments varied. These observations show a difference between the two methods on volatile release, though neither method was successful at giving consistent volatile release of LE and GE EOs over 24 h. Kfoury et al. [23] have found that relative humidity (RH) impacted the release of guest from ICs, with higher humidity increasing the guest release. A significant increase in volatile emission was recorded at 18 h in LG ICs. Samples were stored in a desiccator during periods of time for the controlled release, and exposure to the general laboratory environment while sampling may have led to fluctuating RH levels, which could have led to the faster release of volatiles from the complexes [34].

NMR has proven useful [11,35] to assess IC formation, with ^1^H-NMR differences observed for free and complexed cyclodextrins. The ^1^H-NMR analysis revealed that there was an up-field shift for H-3 and H-5 of β-CD for all the ICs. This indicates that the desired ICs were formed. The EO molecule is located in the lipophilic cavity of the β-CD forming the IC, and its H-3 and H-5 are shielded by intermolecular interactions with the EO, giving more up-field shifts. The change in chemical shifts were largest in GE-2, where the H3 signal changed by −0.038 ppm and the H-5 signal changed by around −0.067. For all three EOs a difference in the magnitude of change in chemical shift was observed between the two preparation methods. This indicates that the method of preparation influenced the success of the IC formation as shown by the NMR data. The interaction between cyclodextrin and the guest molecules in the solid state was assessed through FTIR spectroscopy, evaluating the changes recorded in the ICs compared to the free β-CD and EO. IC formation with all EOs was demonstrated by studying the modification of peak shape, position, and intensity [27]. For all EOs the IC formation changed the FT-IR spectra. The IC spectrums showed prominent bands from β-CD and changes in the characteristic bands of the free EOs. These observations are supported by previous work [36,37] where the presence of hydrogen-bonding and other intermolecular forces indicate the IC formation. The UV-Vis analysis has proven useful to investigate the solubility enhancement of IC formation [38]. The UV-Vis analysis of the ICs, EO, and PM in the solution indicate that ICs were formed, and all had greater solubility in water than PM. The UV data show an increase in absorbance from PM to ICs, and in some incidences the absorbance of ICs was greater than that of the EO alone, indicating an even larger change in the solubility of EO in water. The observed increase in solubility of the encapsulated EOs expand the formulation potential of these active ingredients as a product to repel ticks. The morphological analysis performed through SEM revealed that all the ICs appeared as irregular lamellar particles with smaller particle size compared to the free β-CD. This indicates that some changes in the morphology of β-CD has occurred, but no conclusions about the formation of the IC can be drawn from the SEM images alone. From the SEM analysis LG-1 and LG-2 appear to have the smallest particle size on average. 

ICs exerted a significant repellent activity against *I. scapularis* nymphs. The vertical bioassay was designed to assess the ability of IC to stop the tick from climbing towards the host. Among the selected EOs, GE was the most effective, repelling up to 90% of tested ticks. The ability of ticks to detect the main component of GE essential oil (i.e., β-citronellol) was previously reported [39] which has excellent tick repellent properties [14], and the encapsulation in the cyclodextrin cavity may have improved efficacy and longevity of this effective active ingredient. Interestingly, the GE complex prepared using method 2 was highly effective, while the GE complex from method 1 repelled only 70% of tested ticks. In terms of the releasing profile, the complex prepared with method 2 has a higher volatile emission that may be linked to the better tick repellent activity observed compared to the GE complex prepared according to method 1. The inclusion complex with lemongrass essential oil provided a product with significant tick repellent activity. Complexes prepared with the two different methods exerted the same repellent action (80%), possibly explained by the similar emission profiles recorded in both cases (Figure 3). LG EO is also well-known as an effective tick repellent [13] and the functionalization of the active ingredient through inclusion in the host cavity may provide an effective delivery tool. Surprisingly, the LE complex did not perform as well as expected. This essential oil with 3, 8-*p*-menthane-diol as the main component has been reported to have remarkable repellent properties against blood-feeding arthropods [40,41]. However, the LE ICs prepared in this study were the least effective in repelling ticks. The poor repellent performance may be linked to the lower inclusion efficiency and chemical nature of main guest molecule (e.g., citronellal). It is important to note that the preparation of the bioassay was done using equal amounts of ICs, meaning that the amount of active ingredient is not directly comparable: The apparent lower repellency of LE may be simply a reflection of the lower amount of EO encapsulated. In general, the results from the bioassay indicated that the repellency of the EOs is conserved when they are hosted within β-CD. Further testing on the degree of repellency needs to be performed to corroborate the current results.

Polymers can play an important role in tick and insect repellent development [42]. They are used to coat active molecules for creating a controlled release system that can prolong the repellency of the active ingredient [43,44,45,46]. Recent studies have focused on testing repellents against mosquitos, but not much attention has been devoted on testing polymer based-products against ticks [44,45,47,48], which present a different chemosensory system that may be impacted differently by the encapsulation of active ingredients. [39]. In this study, the production of EO ICs has been performed on a laboratory scale. The synthetic process, with limited use of organic solvents and heat, and relatively low cost of starting materials, may be easily transferred to a large-scale production for further commercial development. The preparation time was an area of focus in this study, and overall a shorter method did not have a significant impact on the product quality.

## 5. Conclusions

In the present study, the encapsulation of selected essential oils with known tick repellent activity with β-CD ICs was successfully achieved. The two co-precipitation methods employed for the synthesis of ICs revealed how the affinity of selected guest molecules impacted the successful encapsulation and the releasing profiles recorded. Lemongrass oil and geranium Egyptian provided the best % IE and lemongrass oil had the most consistent volatile release. Lemon eucalyptus was the least compatible with the host, providing low % IE and inconsistent release profile, an indication that the guest was poorly retained by the β-CD complex. Prepared ICs resulted in being effective in repelling ticks in lab conditions. The inclusion complex with geranium Egyptian essential oil prepared according to method 2 was the most effective, repelling up to 90% of tested ticks, while lemongrass oil-based ICs prepared with the two methods were equally effective in exerting 80% repellent action. These complexes with tick repellent activity can have important applications in the development of novel formulations and devices to protect the public against tick bites. Essential oil-based ICs with documented repellent activity can be used to synthesize functional textiles to obtain the controlled released of the active ingredients [49,50,51]. Our findings indicate that the proposed synthetic approaches are effective in preparing stable essential oil-based ICs. The controlled release of volatile guest molecules makes the ICs ideal substrates to be used in the future for the development of tick repellent products, such as pest repellent-treated textiles. 

## Figures and Tables

**Table 1:** Summary of essential oils and synthetic methods used to prepare β-cyclodextrin inclusion complexes.

Essential Oil	Method	ID
Lemongrass (LG)	1	LG-1
Lemongrass (LG)	2	LG-2
Lemon eucalyptus (LE)	1	LE-1
Lemon eucalyptus (LE)	2	LE-2
Geranium Egyptian (GE)	1	GE-1
Geranium Egyptian (GE)	2	GE-2

**Table 2:** Mass (μg) of essential oil per mg of inclusion complex, and % inclusion efficiency (% IE) of essential oil in the inclusion complexes. Essential oils contained in the inclusion complexes were extracted in triplicate, diluted with internal standard solution in hexanes, and analyzed by GC-FID. Quantification of essential oil was done using 400–10 ng/μL standard curves containing 40 ng/μL internal standard in hexanes. Values were calculated from averaging measurements from three extracts and are presented along with their standard error (SE).

Treatment	Mass Concentration Essential Oil			% IE (±SE)
	μg/mg ± SE	*t*	*p*	
LG-1	65 ± 2	-	-	65 ± 2
LG-2	63 ± 3	0.69	0.978	69 ± 4
LE-1	30 ± 1	-	-	25 ± 1
LE-2	24 ± 1	1.78	0.513	20 ± 1
GE-1	73 ± 5	-	-	72 ± 5
GE-2	88 ± 6	−4.78	0.007	69 ± 4

**Table 3:** Mass (ng) of essential oil (EO) volatiles per mg of inclusion complex ± standard error (SE) released at 0, 3, 6, 9, 18, and 24 h. Inclusion complexes were stored on a watch glass at 10–20% humidity and room temperature for the indicated times. Volatiles released by 10 mg (lemongrass (LG) and geranium Egyptian (GE)) or 5 mg (lemon eucalyptus (LE)) of inclusion complexes in 20 mL vial were analyzed by SPME/GC-MS and quantified using liquid standard curves of respective essential oil in hexanes.

Treatment	EO Volatiles (ng/mg ± SE)							
	0 H	3 H	6 H	9 H	18 H	24 H	*t*	*p*
LG-1	0.40 ± 0.10	0.44 ± 0.06	0.39 ± 0.01	0.47 ± 0.09 *	0.75 ± 0.02	0.41 ± 0.10	-	-
LG-2	0.83 ± 0.26	0.31 ± 0.06	0.31 ± 0.12	0.44 ±0.11	0.71 ± 0.08	0.47 ± 0.04	0.11	1.0
LE-1	189 ± 9	16.8 ± 0.8	10.9 ± 0.7	10 ± 1	8.2 ± 0.4	7.8 ± 0.8	-	-
LE-2	14 ± 3	7.8 ± 0.1	6.7 ± 0.1	6.1 ± 0.4	6.0 ± 0.3	6.3 ± 0.2	−43.4	<0.001
GE-1	0.4 ± 0.1	0.20 ± 0.04	0.20 ± 0.05	0.13 ± 0.05	0.09 ± 0.02	0.08 ± 0.04	-	-
GE-2	5.2± 0.2	3.3 ± 0.4	2.3 ± 0.6	0.76 ± 0.08	1.3 ± 0.2	1.1 ± 0.1	1.2	0.8

* Value calculated from the average of two measurements.

**Table 4:** Chemical shift (δ) and change in chemical shift (Δδ) of H-3 and H-5 of β-cyclodextrin and inclusion complexes. Protons are labeled according to Figure 7 of the repeating unit of β-cyclodextrin.

Treatment	δ H-3 (ppm)	δΔ	δ H-5 (ppm)	δΔ
β-CD	3.9465	-	3.8354	-
LG-1	3.9214	−0.0251	3.7976	−0.0378
LG-2	3.9202	−0.0263	3.7951	−0.0403
LE-1	3.9279	−0.0186	3.8035	−0.0319
LE-2	3.9342	−0.0123	3.8190	−0.0164
GE-1	3.9093	−0.0372	3.7699	−0.0655
GE-2	3.9086	−0.0379	3.7681	−0.0673

**Table 5:** Mean percentage of repelled (± SEM) ticks exposed to different treatments in vertical bioassays (N = 10).

Treatment	Repellency (±SEM)		
	%	Z	*p*
Free β-CD	0.0 ± 0.0	-	-
LG-1	80.0 ± 13.0	−3.53	0.002
LG-2	80.0 ± 13.0	−3.61	0.001
LE-1	50.0 ± 16.0	−2.21	0.05
LE-2	70.0 ± 15.0	−3.15	0.003
GE-1	70.0 ± 15.0	−3.09	0.006
GE-2	90.0 ± 10.0	−4.06	0.0003

## Data Availability

Not applicable.

## Appendix A

^1^H-NMR results. Table A1 provides full assignment of the β-CD protons chemical shift (δ).

**Table A1:** Assigned peaks from 400 MHz ^1^H-NMR of β-CD in D_2_O. Protons are labeled according to Figure 7 of the repeating unit of β-CD.

Proton	δ (ppm)
H-1	5.0492
H-2	3.6278
H-3	3.9465
H-4	3.5642
H-5	3.8354
H-6ab	3.8583

FT-IR results. Table A2 provides a summary of the FT-IR results obtained for better comparison.

**Table A2:** Summary of FT-IR analysis of β-cyclodextrin (β-CD), lemongrass essential oil, (LG oil), inclusion complexes LG-1 and LG-2, lemon eucalyptus essential oil, (LE oil), inclusion complexes LE-1 and LE-2, geranium Egyptian essential oil, (GE oil), geranium Egyptian inclusion complexes GE-1 and GE-2. All values are in cm^−1^.

Band Assignment	β-CD	LG oil	LG-1	LG-2	LE oil	LE-1	LE-2	GE oil	GE-1	GE-2
O-H stretching	3383	-	3356	3356	-	3363	3363	3371	3363	3352
=CH stretching	-	3479	-	-	3443	-	-	-	-	-
–CH_2_ stretching	2939	2966–2858	2924	2924	2920	2924	2924	2962–2874	2924	2924
C=O stretching		1678	1674	1674	1724	1720	1716	1728	-	-
H–O–H bending	1639	-	-	-	-	1639	1639	-	1639	1639
=CH scissoring	-	1446	-	-	1450	-	-	1450	-	-
C–O–C stretching	1157	-	-	-	-	-	-		-	-
